# Characterization of Protective Human CD4^+^CD25^+^ FOXP3^+^ Regulatory T Cells Generated with IL-2, TGF-β and Retinoic Acid

**DOI:** 10.1371/journal.pone.0015150

**Published:** 2010-12-17

**Authors:** Ling Lu, Xiaohui Zhou, Julie Wang, Song Guo Zheng, David A. Horwitz

**Affiliations:** 1 Division of Rheumatology, Department of Medicine, Keck School of Medicine at University of Southern California, Los Angeles, California, United States of America; 2 Department of Liver Transplantation, First Affiliated Hospital of Nanjing Medical University, Nanjing, China; 3 Immune Tolerance Center Shanghai East Hospital, Tonji University of Medicine, Shanghai, China; New York University, United States of America

## Abstract

**Background:**

Protective CD4+CD25+ regulatory T cells bearing the Forkhead Foxp3 transcription factor can now be divided into three subsets: Endogenous thymus-derived cells, those induced in the periphery, and another subset induced ex-vivo with pharmacological amounts of IL-2 and TGF-β. Unfortunately, endogenous CD4+CD25+ regulatory T cells are unstable and can be converted to effector cells by pro-inflammatory cytokines. Although protective Foxp3+CD4+CD25+ cells resistant to proinflammatory cytokines have been generated in mice, in humans this result has been elusive. Our objective, therefore, was to induce human naïve CD4+ cells to become stable, functional CD25+ Foxp3+ regulatory cells that were also resistant to the inhibitory effects of proinflammatory cytokines.

**Methodology/Principal Findings:**

The addition of the vitamin A metabolite, all-trans retinoic acid (atRA) to human naïve CD4+ cells suboptimally activated with IL-2 and TGF-β enhanced and stabilized FOXP3 expression, and accelerated their maturation to protective regulatory T cells. AtRA, by itself, accelerated conversion of naïve to mature cells but did not induce FOXP3 or suppressive activity. The combination of atRA and TGF-β enabled CD4+CD45RA+ cells to express a phenotype and trafficking receptors similar to natural Tregs. AtRA/TGF-β-induced CD4+ regs were anergic and low producers of IL-2. They had potent *in vitro* suppressive activity and protected immunodeficient mice from a human-anti-mouse GVHD as well as expanded endogenous Tregs. However, treatment of endogenous Tregs with IL-1β and IL-6 decreased FOXP3 expression and diminished their protective effects *in vivo* while atRA-induced iTregs were resistant to these inhibitory effects.

**Conclusions/Significance:**

We have developed a methodology that induces human CD4^+^ cells to rapidly become stable, fully functional suppressor cells that are also resistant to proinflammatory cytokines. This methodology offers a practical novel strategy to treat human autoimmune diseases and prevent allograft rejection without the use of agents that kill cells or interfere with signaling pathways.

## Introduction

CD4^+^ regulatory T cells (Tregs) bearing the Forkhead Box P3 (Foxp3) transcription factor are required to maintain immunologic homeostasis and prevent autoimmunity [1], [2]. Mutations of the Foxp3 gene result in immune dysregulation and multiorgan autoimmunity [3]. Both CD4+ cells and CD8+ cells can express Foxp3 [4], [5], but the former have received the most attention. Because abnormalities in the numbers and function of Tregs can lead to autoimmunity, allergy and graft rejection, manipulation of these cells to correct these defects offers a novel treatment strategy [6]. Endogenous CD4^+^Foxp3^+^ cells can be divided into thymus-derived, natural regulatory T cells (nTregs) which constitutively express high levels of CD25, the IL-2 receptor alpha chain and those induced in the periphery from CD4^+^CD25^−^Foxp3^−^ precursors by a TGF-β dependent mechanism (iTregs). In mice and humans these two subsets have been indistinguishable phenotypically until recently [7], and may have separate or synergistic roles *in vivo* [8], [9]. In humans CD4^+^FOXP3^+^ Tregs express high levels of CD25 and low levels of CD127, the IL-7 receptor alpha chain [10].

In addition to endogenous Foxp3+ Tregs, substantial evidence exists that the combination of IL-2 and TGF-β can induce naïve CD4^+^CD25^−^ cells to become FOXP3^+^ iTregs in both mice and humans. In mice, suboptimal polyclonal TCR stimulation of naïve CD4^+^ cells with IL-2 and TGF-β can induce iTregs that have protective effects in autoimmune diabetes [11], experimental autoimmune encephalitis[12] and myasthenia gravis [13]. Because of decreased numbers and/or function of FOXP3^+^ Tregs in human autoimmune diseases [14], the transfer of iTregs generated *ex-vivo* could be therapeutic to subjects with these diseases.

In humans CD4^+^CD25^−^ cells activated by either superantigens or alloantigens with IL-2 and TGF-β developed potent *in vitro* suppressive activity [15], [16], and these alloantigen-induced FOXP3^+^ iTregs could also induce other CD4^+^CD25^−^ cells to become TGF-β dependent suppressor cells [17]. One group recently also reported that polyclonal TCR stimulation of naïve CD4^+^ cells with TGF-β could result in FOXP3^+^ suppressor cells [18]. However, the generation of fully functional polyclonal human FOXP3^+^ iTregs *ex vivo* is controversial. First, TCR activation without TGF-β can induce naive CD4^+^ cells to transiently express FOXP3 [19]. Secondly, although we and others have observed that TGF-β can greatly increase FOXP3 expression and stability, after one week *in vitro* suppressive activity of these human CD4^+^ cells was not greater than control cells [20], [21]. Moreover, unlike nTregs which are anergic in response to TCR stimulation, these human CD4^+^ cells primed with TGF-β produced IL-2 and proliferated robustly following re-stimulation. Interestingly, however, repeated stimulation of TGF-β primed CD4^+^ cells did result in anergy, membrane-expression of TGF-β, and *in vitro* suppressive activity similar to that described with nTregs [20], [22]. We concluded that human TGF-β primed CD4^+^ cells one week after culture were partially differentiated cells and required a much longer time to mature than similar mouse Foxp3^+^ iTregs [21]. Thus, agents that accelerate cell differentiation might be useful for a more rapid generation of human iTregs *ex-vivo*.

Retinoic acid (RA), a vitamin A derivative, has an important role in the development of various organs including the immune system. RA metabolites strongly contribute to the maintenance of immunologic tolerance. All-*trans* retinoic acid (atRA), an active metabolite of retinoic acid, markedly enhances TGF-β-induced Foxp3 expression and stability in mice [23], and the expansion of these iTregs by either direct cytokine-dependent [24] or cytokine independent mechanisms [25]. In human CD4^+^ cells, atRA has been reported to induce histone acetylation at the FOXP3 gene promoter and expression of the FOXP3 protein [26]. Recently, atRA has been shown to enhance the stability and expansion of TGF-β induced iTreg and endogenous nTreg cells [27]. Here we have extensively characterized the phenotype and functional properties of iTregs induced by TGF-β and atRA. We report that atRA markedly accelerates the differentiation of naïve cells to fully functional suppressor cells. Unlike CD4^+^ cells generated in one week with IL-2 and TGF-β the presence of atRA during this time enabled naïve CD4^+^ cells to demonstrate strong protective suppressive effects not only *in vitro*, but also *in vivo* when transferred to immunodeficient mice. Moreover, unlike endogenous nTregs, atRA-induced iTregs were resistant to the inhibitory effects of IL-1β and IL-6. Thus, we have demonstrated that it is possible to induce naïve human CD4^+^ cells to rapidly become iTregs that have protective effects *in vivo* and that are resistant to the inhibitory effects of proinflammatory cytokines.

## Results

**Figure 1:**
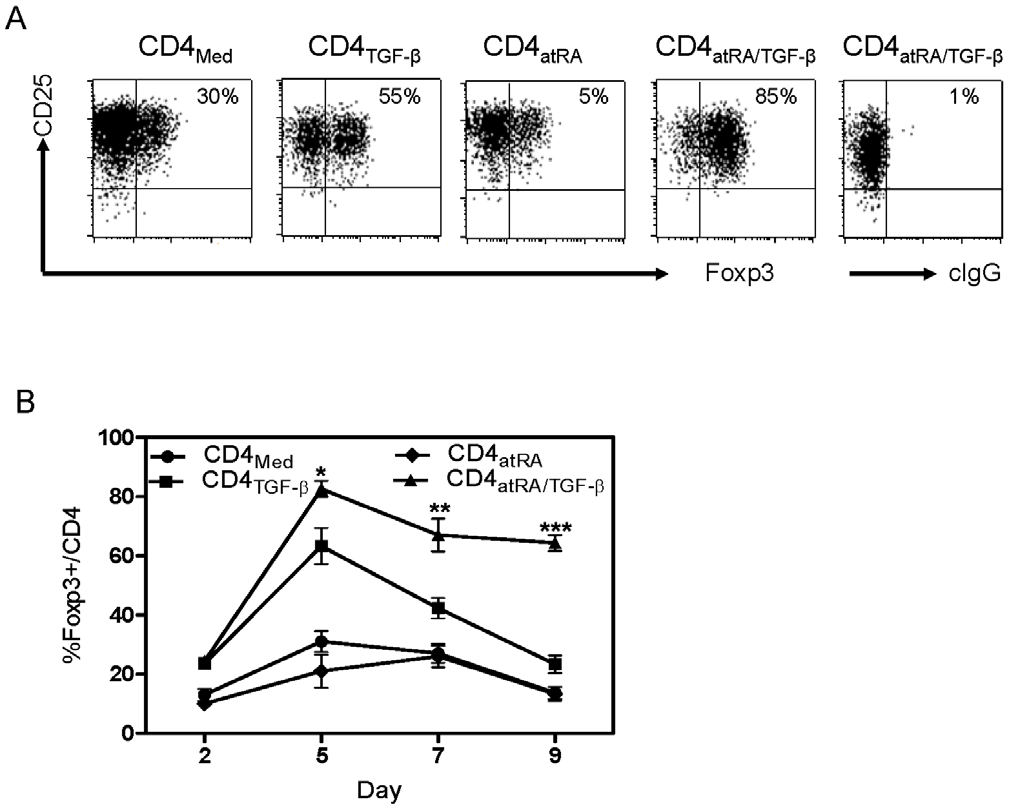
AtRA enhances and stabilizes FOXP3 induced by TGF-β. CD4^+^CD45RA^+^ cells were stimulated with suboptimal numbers of antiCD3/CD28 beads and IL-2, with and without TGF-β and atRA. (A) Representative expression of CD25 and FOXP3 by flow cytometry after 5 days of culture. (B) Stability study: Naive CD4 cells were similarly activated with IL-2 (50 U/ml) with and without TGF-β and atRA. At day 5, the medium was removed and the additives replaced with IL-2 (20 U/ml), an amount that is not sufficient to sustain FOXP3 induced by IL-2 and TGF-β. At the various days indicated, the percentage of FOXP3^+^ cells is indicated for each conditioned CD4^+^ subset studied. The values indicate the mean ± SEM of 4 separate experiments.

The addition of atRA to TGF-β enhanced FOXP3 stable expression and accelerated the maturation of naïve CD4^+^ cells to memory/effector regulatory cells. Naïve CD4^+^ cells were activated with suboptimal anti-CD3/28 beads titrated to numbers needed for the cells to express CD25. While IL-2 and TGF-β increased the percentage of CD4^+^CD25^+^ cells that expressed FOXP3 after 5 days of culture, the addition of atRA to TGF-β markedly enhanced this effect (**Figure 1** **, A and B**). Time course studies revealed that adequate levels of IL-2 can sustain TGF-β induced FOXP3 [28] (result not shown). However, with less IL-2, TGF-β induced Foxp3 also decreased after culture for 7 to 9 days, while FOXP3^+^ cells induced by atRA and TGF-β remained stable (**Figure 1B**). This finding is in agreement with Wang et al [27]. Thus, the combination of atRA and TGF-β rapidly induced naïve CD4^+^ cells to express FOXP3, and the stability of this transcription factor is less IL-2 dependent than FOXP3 induced by TGF-β alone.

**Figure 2:**
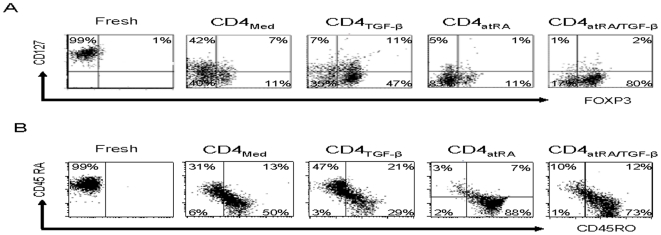
AtRA accelerates the maturation of naïve CD4^+^ cells to effector/memory cells. CD4^+^CD45RA^+^CD25^−^naive T cells were cultured in the presence and absence of atRA (0.1 µM) and/or TGF-β-1 (5 ng/ml) with IL-2 (50 U/ml) for 5 days. As indicated by flow cytometry: (A) By itself, atRA increased the down-regulation of CD127, and (B) the transition from CD45RA to CD45RO. In combination with TGF-β, atRA markedly increased the proportion of FOXP3^+^ cells that became CD127^dim^ and CD45RO^+^. The result is representative of four separate experiments.

Although IL-7 is an important growth and survival factor for certain T cell subsets, CD127, the α chain of the IL-7 receptor, is down-regulated on nTreg cells. These cells are CD25^+^CD127^dim^ [10]. **Figure 2A** shows that following activation for 6 days, CD127 displayed by naïve CD4^+^ cells was moderately downregulated. While TGF-β enhanced this down-regulation of CD127, atRA resulted in more than 90% of the CD4^+^ cells becoming CD127^dim^. However, only a small fraction expressed FOXP3. Because >80% of CD4^+^ cells activated with atRA and TGF-β expressed FOXP3, presumably most were also CD127^dim^. Similarly, although atRA alone also accelerated transition from CD45RA^+^ to CD45RO^+^ cells, most of these cells were Foxp3^−^. Only with the combination of atRA and TGF-β do most CD45RO^+^ cells also express FOXP3 (**Figure 2B**).

**Figure 3:**
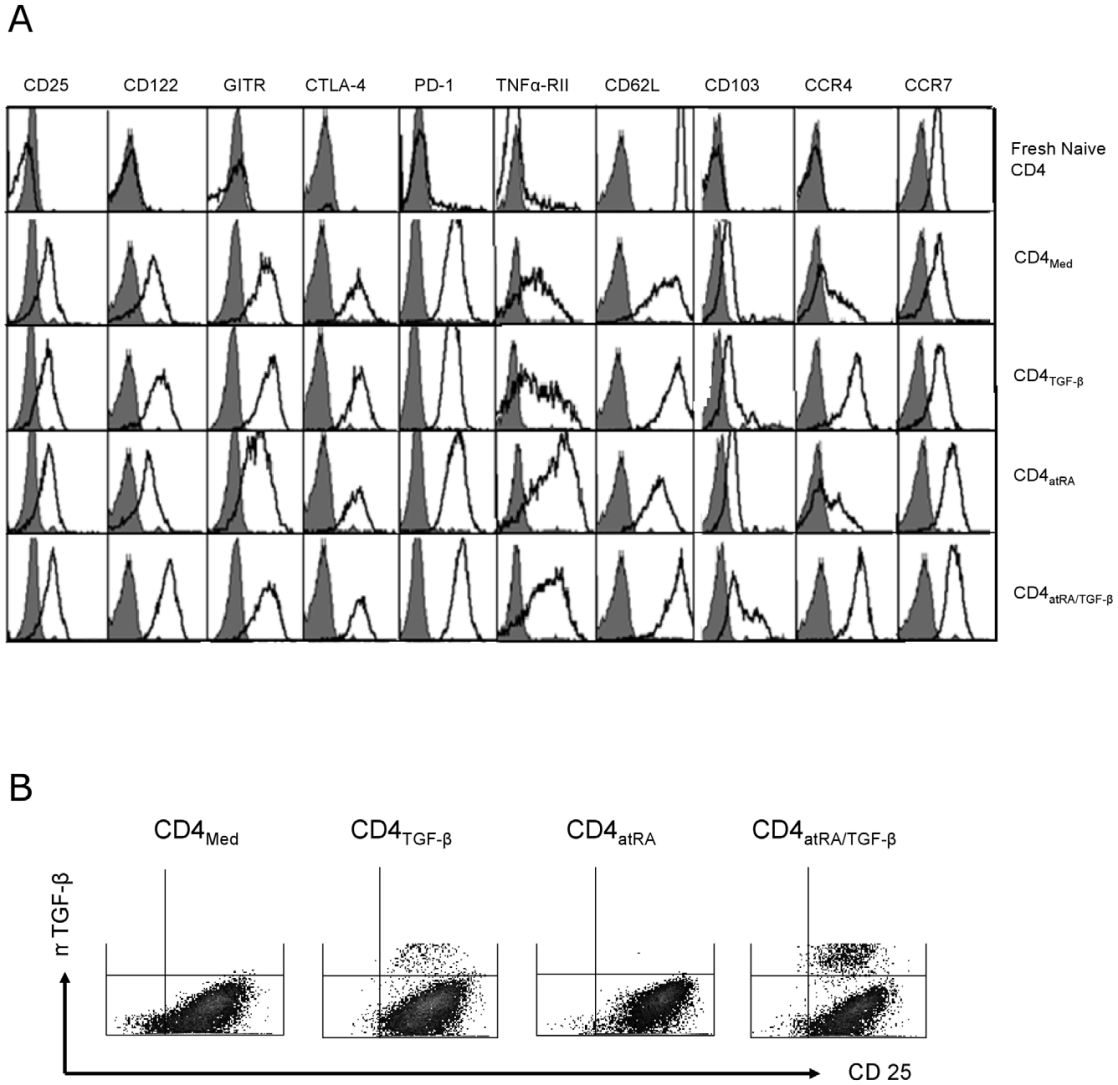
Phenotypic characterization of human atRA/TGF-β induced CD4^+^ iTregs at day 5. Naïve CD25 depleted CD4^+^ cells were stimulated with CD3/CD28 beads and the additives indicated above for 5 days. (A) The staining intensity of each marker and the isotype control is shown in comparison with the staining of unstimulated CD4 naïve cells. (B) The cells were rested in fresh medium containing 10% FCS for 24 hours and restimulated with anti CD3/CD28beads (1∶1) and IL-2 (20 U/ml) for 48 hours. FACS analysis of CD25 and mTGFβ1 expression by the various CD4^+^ conditioned subsets is shown. The results are representative of three separate experiments.

**Figure 3A** shows ten markers characteristically expressed by nTreg cells [22], [29]. Of these, naïve CD4^+^ cells express only L-selectin (CD62L) and CCR7 (see below). The figure shows that following TCR activation with atRA and TGF-β added separately or together, naïve CD4^+^ cells acquire other Treg-related receptors and retain CD62L and CCR7. These cells expressed GITR, and CTLA-4, although these markers are also expressed by control activated T cells. Besides FOXP3 and CD127 shown above, the combination of atRA and TGF-β increased the intensity of CD122, PD-1, and TNFRII (Tumor necrosis factor receptor II) staining. Although some activated T-_Med_ expressed TNFRII^+^, atRA markedly enhanced this effect. These cells, however, were FOXP3^−^. Only with both atRA and TGF-β did most of these cells display FOXP3. TNFRII expression has been described on mouse nTregs, but to date not on human Tregs [29].

Finally, activated nTreg cells express membrane-bound TGF-β [30]. We observed that adding atRA to TGF-β greatly increased the number of iTreg cells expressing membrane TGF-β (**Figure 3B**). Unlike most markers that are stained at 4°C, membrane-bound TGF-β is maximal at 37°C. Thus, the addition of atRA to TGF-β increases the conversion of activated CD4^+^ cells to the effector/memory cells and accelerates their phenotypic differentiation to FOXP3^+^ Treg cells.

Activation of naïve CD4^+^ cells with atRA added to TGF-β enables them to retain or acquire receptors needed to recirculate from blood to lymphoid organs. Naïve and central memory CD4^+^ cells constitutively express the lymphoid homing receptors CD62L and CCR7 that enable them to circulate from blood to secondary lymphoid tissues [31]. CD4^+^CD45RO^+^ effector cells generally lack these receptors and express others that enable them to migrate to extravascular sites. Even though most nTregs are CD45RO^+^, they continue to express CD62L and CCR-7 which facilitate their trafficking to lymphoid organs. CCR7 has been reported to be needed for nTreg homing and function in lymphoid tissues [32]. **Figure 3A** shows that following activation of naïve CD4^+^CD45RA^+^ cells, down-regulation of CD62L is decreased by TGF-β. Similarly, activation with atRA decreases CCR7 downregulation. Accordingly, when naïve CD4^+^ cells were activated with both TGF-β and atRA, expression of both CD62L and CCR7 was retained, even though the cells they underwent transition from CD45RA to CD45RO (**Figure 2B**). Thus, similar to nTregs, most atRA/TGF-β-induced iTregs are CD45RO^+^ effector/memory cells that continue to express CCR7 and CD62L.

Human nTregs also express CCR4, another lymphoid homing receptor [33]. Interestingly, TGF-β also induced naïve CD4^+^ cells to express CCR4 (**Figure 3A**). CD103 (αE integrin) is another homing receptor especially important in the mucosal immune system [34]. Human nTregs and iTregs express CD103 [18]. Here we observed that the combination of TGF-β and atRA induced higher levels of CD103 than either agent used alone (**Figure 3A**). In other experiments, atRA/TGF-β-induced iTregs and expanded nTregs were restimulated with anti-CD3/28 beads and low dose IL-2 for 3 days. We observed that CCR4 and CCR7 expression remained high on iTregs, but began to decrease on nTregs (**Figure S1**).

**Figure 4:**
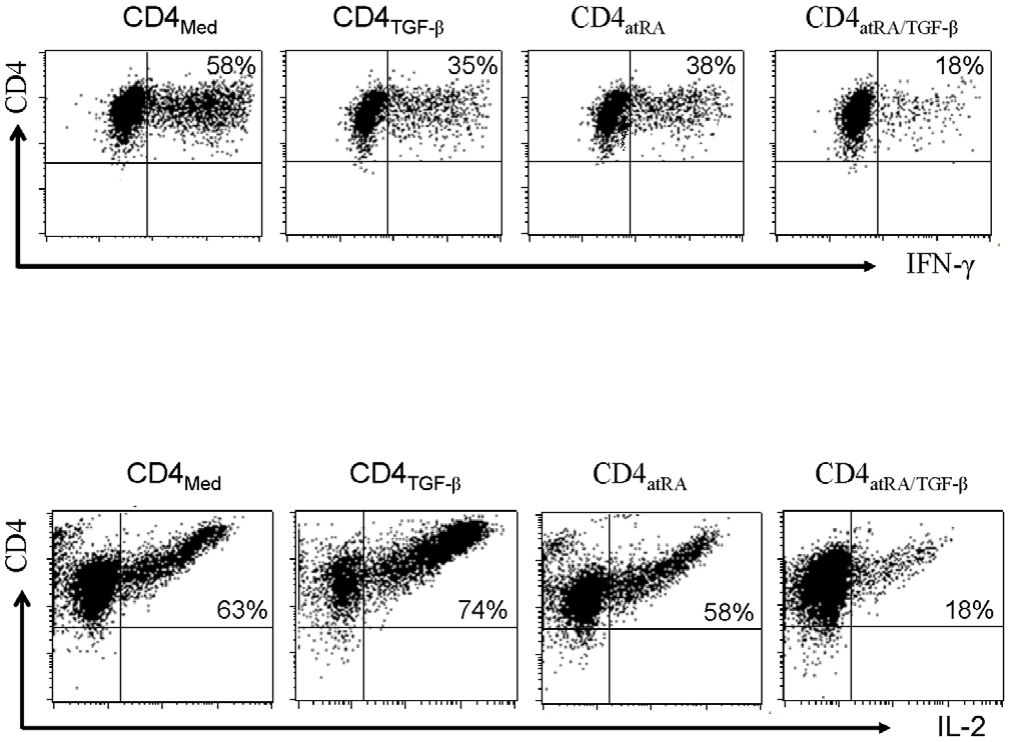
AtRA/TGF-β-primed CD4^+^ cells produce less IL-2 and IFN-γ. Naïve CD4^+^ cells were stimulated with the additives described above for 5 days, washed and the beads were removed. The cells were rested for 24 hours and then restimulated with anti CD3/CD28beads for 48 hours. PMA and ionomycin was added for the last 5 hours, and brefeldin A for 4 hours, and intracellular IL-2 and IFN-γ cytokine expression was then assessed by flow cytometry. The results shown are representative of three separate experiments.

**Figure 5:**
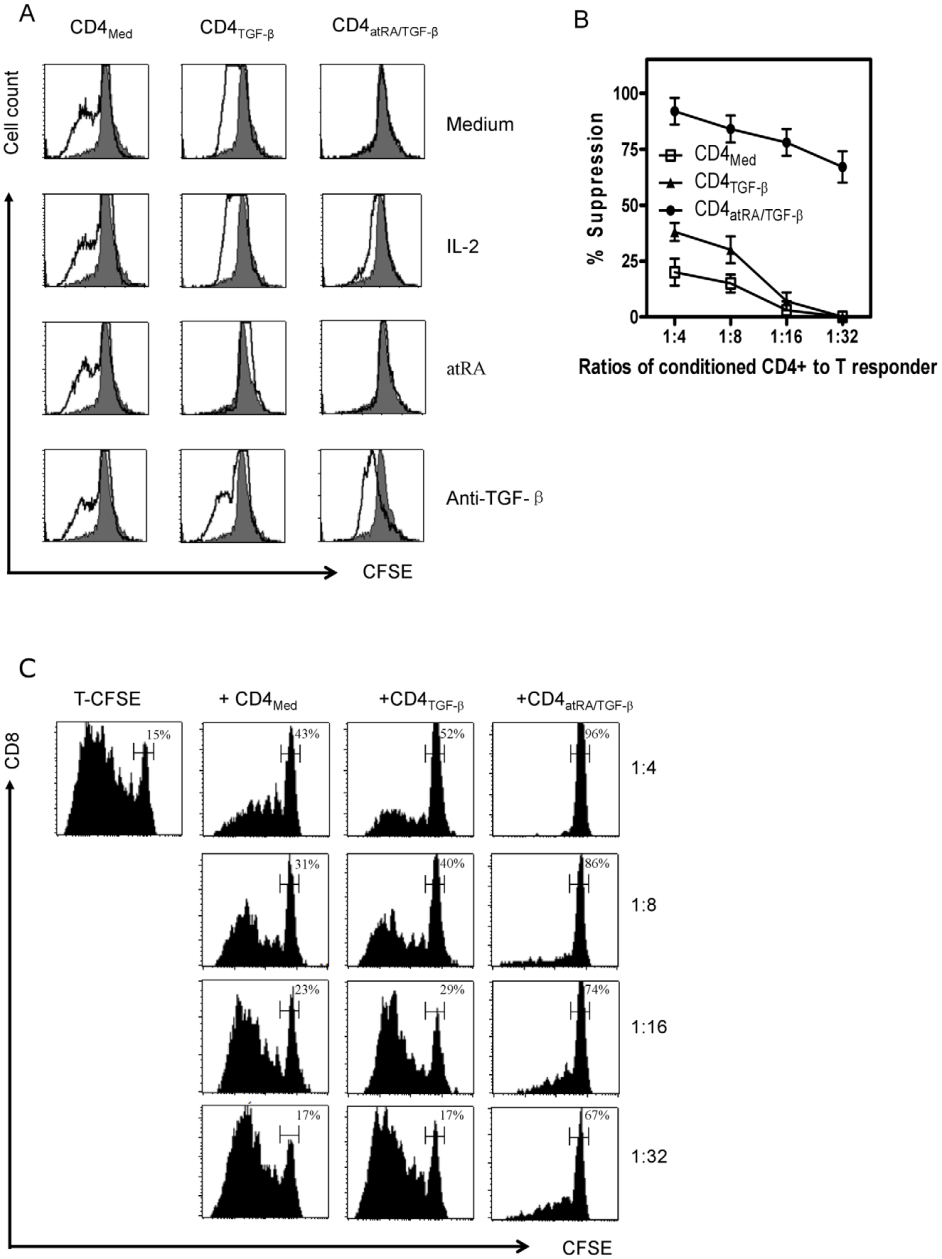
CD4^+^CD25^+^Foxp3^+^ cells induced by atRA and TGF-β are anergic and have potent suppressive effects in vitro. (A) The various primed CD4^+^ cell subsets indicated were prepared as described above. After culture for 5 days, the cells were washed, rested for 48 hours, and labeled with CFSE. The cells were then restimulated with anti-CD3/CD28 beads with the additives indicated for three days and proliferation was evaluated by CFSE dilution. Note that the addition of atRA to TGF-β primed cells also resulted in anergy, and the hypoproliferative state was abolished by anti- TGF-β. (B) To examine the suppressive capacity of T-Med, T-TGF-β and T-atRA+TGF-β, these primed CD4^+^ cells were mixed with autologous CFSE-labeled T cells in different ratios and stimulated with soluble anti-CD3 (1∶500) with irradiated non T cells as APC (1∶1). At day 4, inhibition of proliferation (CFSE dilution) of responder T cells was analyzed by flow cytometry. The mean ± SEM percent suppression at the various ratios of 3 separate experiments is shown. (C) In this representative experiment, the cells were stained for anti-CD8 and the suppressive activity of various primed CD4^+^ cells subsets on CFSE-labeled CD8^+^ at various Tsuppressor to T effector ratios is shown.

Tregs generated with atRA and TGF-β produce low levels of proinflammatory cytokines, are hypoproliferative *in vitro*, and develop potent suppressive activity *in vitro* and *in vivo*. Unlike naïve CD4^+^ cells primed with TGF-β, the addition of atRA to TGF-β resulted in functional activities similar to those of nTregs within 5 to 7 days following activation. As shown in **Figure 4**, atRA/TGF-β-induced Tregs (iTregs) produced only small amounts of intracellular IL-2 and IFN-γ. They were non-responsive following restimulation with anti-CD3/28 coated beads and remained hyporesponsive (**Figure 5A**). Interestingly, the addition of anti-TGF-β to the cultures restored their proliferative response, a finding suggesting that membrane-bound TGF-β may contribute to anergy *in vitro*.

As has been reported previously, human polyclonally-stimulated CD4^+^ cells primed with IL-2 and TGF-β did not acquire marked suppressive activity [20]. By contrast, CD4^+^ cells primed with atRA and TGF-β developed potent suppressive activity. Marked *in vitro* suppressive activity remained even when iTregs comprised only 3 per 100 T responder cells (1∶32) (**Figure 5** **, B and C**). While most workers report that the *in vitro* suppressive activity of human nTregs is contact-dependent and cytokine independent [20], [22], we also found that the suppressive activity of iTregs was contact-dependent, but in some experiments anti-TGF-β markedly inhibited suppressive activity (**Figure S2**).

**Figure 6:**
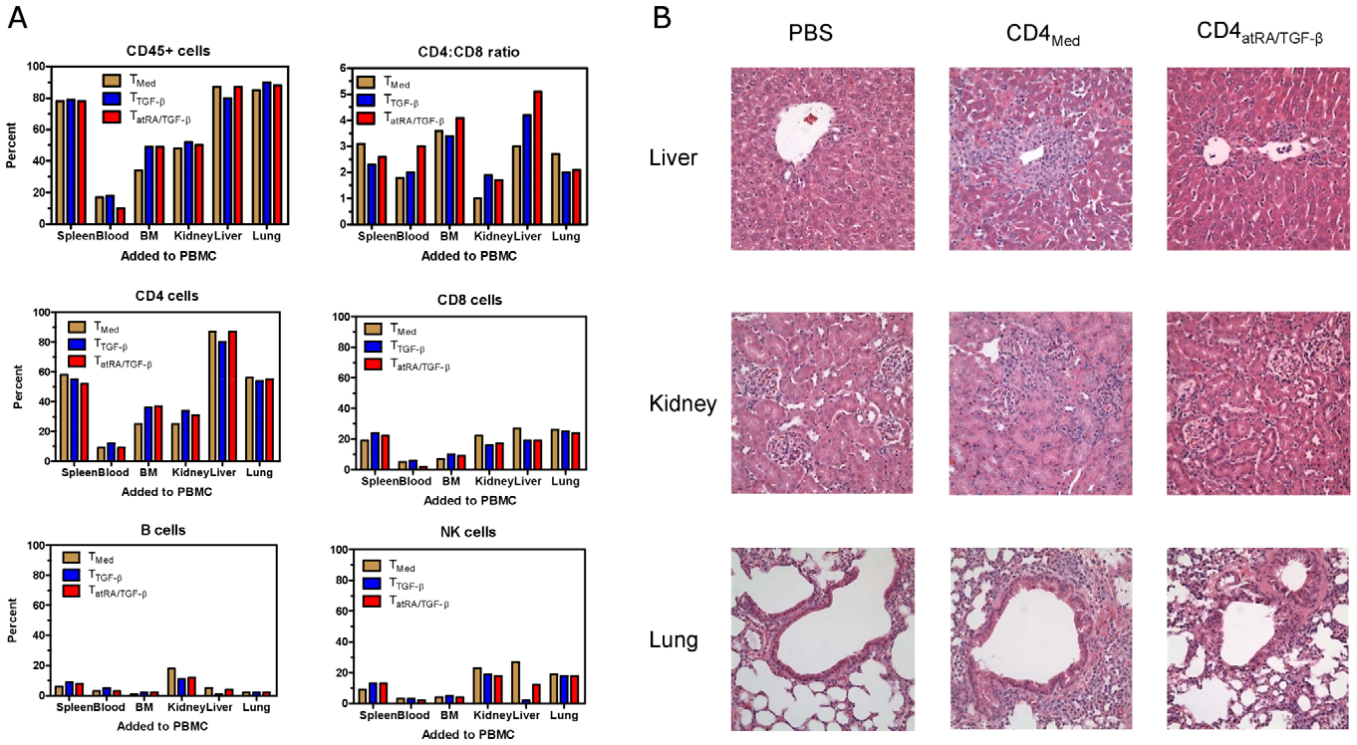
Engraftment human cells in NOG mice 15 days after transfer activated CD4^+^ cells and atRA/TGF-β induced Treg cells. A) Pattern of engraftment: Total human mononuclear cells in the blood, spleen and in collagenase suspensions of liver, kidneys and lungs are indicated as percent human CD45^+^ cells by flow cytometry. CD4/CD8 ratios, CD4, CD8, B cells, and NK cells, also calculated as percent human CD45^+^ cells. B) Hematoxylin-eosin stained sections of liver, kidneys and lungs of mice treated as indicated compared with control mice injected with PBS. The result is representative of studies in four mice.

Finally, since suppressive activity *in vitro* may not correlate with activity *in vivo* [30], we have established a mouse model to examine *in vivo* effects of human iTregs. Lightly irradiated NOD SCID common γ chain^−/−^ (NOG) mice injected with 20 million human CD25 depleted PBMC rapidly lost weight and survived only 14 days. The addition of 5 million naïve CD4^+^ cells, or CD4^+^ cells activated with IL-2 (Tcon) to PBMC resulted in similar demise. Examination of the blood and spleen of mice at 14 days revealed marked engraftment of both human CD4^+^ and CD8^+^ T cells (**Figure 6A**). Examination of other organs demonstrated both extensive mononuclear infiltrates especially in the liver and lung (**Figure 6B**). As reported by Ito and co-workers[35], unlike the transfer of human PBMC to Rag−/− SCID cγ ^−/−^ mice, human PBMC did not migrate to the NOG gut or skin (result not shown). Nonetheless, they had developed the human anti-mouse graft-versus-host disease (GVHD) described by others [35], [36], [37]. The addition of human CD4+ cells primed with IL-2 and TGF-β (T-_TGF-β_) also could not protect mice from this rapidly fatal xeno-GVHD. The mononuclear cell infiltrates and tissue damage observed in these mice was equivalent to mice that received PBMC and T control cells (T-_Med_) (**Figure S3**).

**Figure 7:**
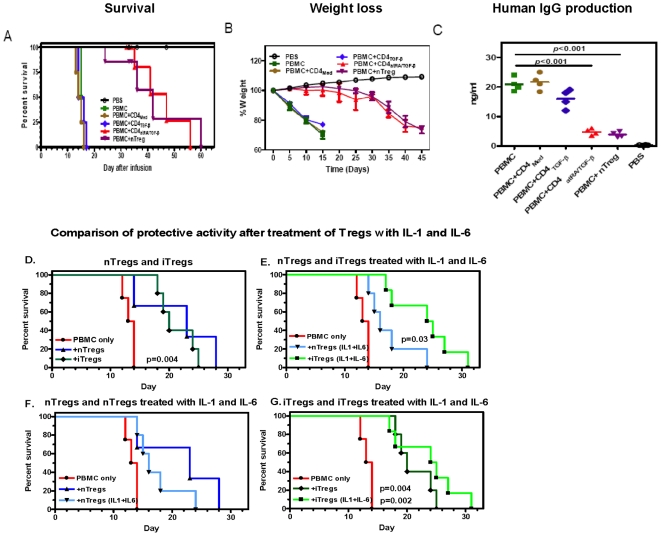
AtRA/TGF-β iTregs have equivalent protective effects in vivo as expanded nTregs and are also resistant to the inhibitory effects of IL-1β and IL-6. A rapidly fatal xenogenic GVHD was induced by the transfer of human PBMC to NOG mice (See materials and methods). Various conditioned CD4^+^ cells cultured for 5 to 6 days were rested for 24 h. Then 5 million were added to 20 million human PBMC and transferred IV to sublethally irradiated NOG mice. Two experiments were combined so that each group contained six to eight mice. A) Survival: TatRA/TGF-β cells significantly enhanced the survival of NOG mice (P<0.01, Log Rank test), B) prevented weight loss (P<0.01): C) Suppressed human IgG production (P<0.01). Panels D–G, Effects of IL-1β and IL-6 on Treg protective effects. Induced Tregs and expanded nTregs were restimulated with IL-1β and IL-6 (See Methods) and 4 million cells were mixed with 20 million PBMC and injected IV into NOG mice. D) Equivalent effects of restimulated iTregs and nTregs; E) Significantly decreased protective effects of nTregs after treatment with cytokines; F) Comparison of nTregs restimulated ± IL-1β and IL-6. G) Comparative effects of iTregs restimulated ± IL-1β and IL-6. Results are a combination of two experiments with 6 mice per group. P values shown were calculated using the Log Rank test.

By contrast, the onset of GVHD was significantly delayed in mice that had received PBMC with iTregs, or expanded endogenous Tregs (nTregs). They did not begin to lose weight until 2 weeks later and survived up to 8 weeks (**Figure 7** **, A and B**). To compare engraftment of human cells in these mice with controls that died at two weeks, other experiments were performed where mice that received PBMC with iTreg or nTregs were also sacrificed at 14 days. **Figure 6B** **(and Figure S3)** shows markedly fewer numbers of mononuclear cell infiltrating the liver and kidneys, although there were peribronchial and a moderate interstitial mononuclear cell infiltrate in the lungs. This infiltration was probably a consequence of the intravascular trafficking of the transferred cells through the lungs.

Interestingly, although Tregs limited the magnitude of engraftment, the percentage of human cells found, and the proportion of CD4, CD8, NK and B cells was similar to the transfer of human PBMC and Tcon cells (**Figure 6A**). The percentage of human CD45^+^ cells present in the blood was small, but comprised >80% of the mononuclear cells isolated from the spleen, liver and lung. In all organs studied engraftment of CD4^+^ cells was greater than CD8^+^ cells. An even greater predominance of CD4^+^ cells was observed in the blood and liver of animals that received iTreg cells. Relatively more NK cells found in spleen, liver, lung and liver compared to blood and bone marrow. The kidney was the only organ with >10% B cells. Thus, to increase the survival of these immunodeficient mice, it is likely that iTreg cells inhibit the magnitude of mononuclear cells trafficking to various organs, but not the pattern of human engraftment. In addition to using protection from GVHD to assess suppressor cell activity *in vivo*, we used suppression of T cell-dependent IgG production. By two weeks after transfer of human PBMC >2 mg/ml of human IgG was detected in mouse serum (**Figure 7C**). The addition of iTregs or nTregs to PBMC markedly suppressed human IgG production.

Because of the possible therapeutic potential of Treg cells generated and expanded *ex-vivo*, they should be not only functional, but also resistant to T effector cell conversion *in vivo*. The plasticity of Foxp3^+^ nTregs has become evident with proinflammatory cytokines such as IL-1β and IL-6 converting human nTreg cells to Th17 cells [38], [39]. These cytokines also inhibit nTreg suppressor cell activity [40]. Accordingly, both atRA-induced iTregs and expanded nTregs were treated similarly with these cytokines. They were restimulated with anti-CD3/28 beads, low dose IL-2 ± IL-1β and IL-6 for three days (see methods). These treated Treg cells were mixed with autologous PBMC and injected into NOG mice. The xeno-GVHD observed in two experiments was even more aggressive than the previous study (maximum survival 14 vs. 18 days). Nonetheless, **Figure 7D** shows that both iTregs and nTregs had significant protective effects (p = 0.004). However, after treatment with IL-1β and IL-6, the protective activity of the atRA-induced iTregs was significantly greater than nTregs (**Figure 7E**). While the protective activity of cytokine-treated nTregs decreased in comparison with control nTregs, the protective activity of cytokine-treated iTregs was modestly greater than control cells (**Figures 7F, G**).

Phenotypic analysis of these Treg subsets after restimulation using low dose IL-2 revealed decreased expression of Foxp3 by nTregs compared with iTregs. PD-1, GITR, CD103, CTLA-4, and CD62L expression by nTregs also decreased and was weaker than iTregs (**Figure S4**). This was probably a consequence of the strong TCR stimulation and high IL-2 dose used for Treg expansion. After iTregs and nTregs were restimulated with IL-1β and IL-6, Foxp3 expression by the nTregs decreased even further. Surprisingly, expression of PD-1, GITR, was greater on iTregs restimulated with IL-1β and IL-6 than iTregs restimulated without these cytokines. Thus, atRA-induced iTregs were not only resistant to the inhibitory effects of IL-1β and IL-6, but these proinflammatory cytokines appeared to have a mild positive effect in stabilizing their phenotype and functional activity.

## Discussion

We have shown that within one week the addition of atRA to IL-2 and TGF-β can induce polyclonally activated human naïve CD4^+^ cells to become CD25^+^FOXP3^+^ Tregs that resemble nTregs phenotypically and functionally. These atRA/TGF-β iTregs have strong suppressive activity both *in vitro* and *in vivo*. While IL-2 and TGF-β can induce polyclonally activated human CD4^+^ cells to express FOXP3, with one exception [18], most workers have found that these cells lack the functional profile of iTreg [20], [21]. However, the addition of atRA to IL-2 and TGF-β completes the maturation of these partially differentiated cells and enables them to protect immunodeficient mice from a xeno-GVHD at least as well as expanded human nTreg cells. Thus, using conventional cell separation methods to separate naïve CD4+ cells from other PBMC, we have shown that these cells can rapidly be induced to become potent suppressor cells.

This study confirms and extends the report by Wang and co-workers showing that atRA enhanced and stabilized TGF-β induced Foxp3 expression and induced naïve human CD4+ cells to express markers also expressed by Treg cells [27]. In humans Broxmeyer's group reported that atRA could induce human cord blood, but not mouse CD4^+^ cells to become FOXP3^+^ suppressor cells without the addition of TGF-β [26]. Herein, we used adult peripheral blood CD45RA^+^ CD4^+^ cells and found that atRA, by itself, could not induce CD4^+^ cells to express FOXP3. In agreement with Wang et al. we found that atRA greatly enhanced the proportion of cells that TGF-β induced to express Foxp3[27]. We also found that TGF-β and atRA could act separately or together in inducing naïve CD4^+^ cells to phenotypically resemble endogenous CD25^+^FOXP3^+^ Tregs, and these effects are summarized in **Table 1**. TGF-β, by itself, enhanced the expression of CD122, the IL-2Rβ chain, GITR, and membrane-bound TGF-β, all markers expressed by nTreg effector cells [22], [30]. AtRA, by itself, enhanced the intensity of, TNFRII and CCR7 expression. The principal effect of atRA, however, was to accelerate the maturation of naïve CD4^+^ cells to become effector/memory cells. Upregulation of CD45RO and down-regulation of CD127 was markedly accelerated. Although these CD127^dim^ cells were FOXP3^−^, in combination with TGF-β, most now co-expressed FOXP3. Moreover, expression of membrane-bound TGF-β was maximal in the presence of atRA and TGF-β. Most suppressive FOXP3^+^ cells are CD127^dim^ [10]. When added together, atRA and TGF-β also enhanced expression of PD-1 and CD103. Thus, atRA and TGF-β have induced human naïve CD4^+^ cells to become phenotypically fully mature Tregs in one week.

**Table 1:** Human naïve CD4^+^ cells polyclonally activated with TGF-β and retinoic acid rapidly become CD25^+^ cells phenotypically similar to nTreg cells.

Relative expression by naïve CD4^+^ cells following TCR activation with:				
nTreg markers	medium only	TGF-β	atRA	Both
Foxp3	+/−	+	+/−	++
CD127dim	+/−	+	+	++
CD45RO	+	+/−	++	+
CD122	+/−	+	+/−	++
CTLA-4	+	++	+	++
GITR	+/−	+	+/−	+
Membrane-bound	-	+	-	++
TGF-β				
CD62L	Decreased	Sustained	Decreased	Sustained
CD103	+/−	+	+/−	++
CCR4	+	++	+	++
CCR7	Decreased	Decreased	Sustained	Sustained

Naïve CD4^+^ cells were stimulated with suboptimal numbers of anti-CD3/28 beads with IL-2 (50 U/ml) in serum-free medium without APCs for 5 days with the indicated additives. The markers were assessed by flow cytometry.

- no significant change.

+/− minimal to modest enhancement.

+ moderate enhancement.

++ marked enhancement.

Both TGF-β and atRA have well established effects on CD4^+^ cell trafficking. TGF-β induces CD103, αE integrin [41], [42], and atRA induces CCR9 and integrin β7 [26]. We found the combination of atRA and TGF-β enhanced CD103 expression by human CD4^+^ cells, as previously observed in mice [42]. We also found that TGF-β induced CCR4 and that atRA enhanced this effect. While human CD4^+^CD25^+^FOXP3^+^ cells expressing CCR4 are found in certain tumors and in rheumatoid synovial tissue [43], to our knowledge this is the first description of CCR4 expressed by iTregs induced *ex-vivo*.

Naïve CD4^+^ cells constantly recirculate from the blood to lymphoid organs and these cells express CD62L (L-selectin) and CCR7 for this function. CCR7 enables CD4^+^ cells to enter lymph nodes through high endothelial venules [32]. Following strong TCR stimulation, these receptors are down-regulated as CD4^+^ cells become CD45RO^+^ effector/memory cells. However, although most nTregs have become CD45RO^+^, they retain both CD62L and CCR7. TGF-β inhibited the loss of CD62L and atRA blocked the downregulation of CCR7 so that the combination of both agents resulted in iTregs similar to nTregs that were effector/memory cells that had not downregulated expression of both of these homing receptors.

In addition to phenotypic similarities, there were several other similarities between iTregs and nTreg cells: 1) As has been described with activated mouse nTregs [44], activated iTregs also express membrane-bound TGF-β. Recently, others reported that human macrophage-induced FOXP3^+^ iTregs express membrane-bound TGF-β [45]. Previously, we had observed that CD4^+^ cells primed with IL-2 and TGF-β had to be repeatedly stimulated before they expressed membrane-bound TGF-β [21]; 2) Both nTregs and iTregs produce much less IL-2 and IFN-γ than conventional CD4^+^ cells. 3) Both Treg subsets proliferate poorly in response to TCR stimulation *in vitro*. However, this property of iTregs was abolished by antagonizing TGF-β, a result suggesting that the membrane-bound TGF-β contributed to this effect. Reversal of nTreg anergy by neutralizing TGF-β is unusual. The anergy experiments also revealed that atRA and TGF-β do not have to be added together for the cells to become hyporesponsive. Although CD4^+^ primed with TGF-β respond robustly to re-stimulation, adding atRA to these cells resulted in anergy.

This is the first report showing that the addition of atRA to IL-2 and TGF-β enabled polyclonally TCR-stimulated naïve CD4^+^ cells to acquire protective suppressive activity *in vivo* within one week. Whether human TGF-β induced CD4^+^CD25^+^FOXP3^+^ cells develop suppressive activity has been controversial [18], [20]. As stated above, we and others could not induce TGF-β primed human naïve CD4^+^ cells to resemble nTregs in one week, However, since we observed that they did acquire these properties following repeated stimulation, we suspected that at one week they were only partially differentiated cells. With the addition of atRA to accelerate maturation, here we document almost complete suppression of T cell proliferation with only 1 iTreg added to 32 T responder cells, and *in vivo* suppressive activity at least as strong as nTreg cells. We also assessed the ability of iTreg cells to block a xeno-GVHD and prevent human T cell-dependent IgG production in NOD SCID IL-2R common γ chain^−/−^ immunodeficient deficient (NOG) mice. Others have used RAG^−/−^ SCID cγ chain^−/−^ mice to induce a human xeno-GVHD, and demonstrated protective effects of expanded endogenous nTregs [36], [37]. To induce GVHD these mice must be irradiated and given toxic chlordronate liposomes to deplete macrophages. As documented by others, to develop GVHD in NOG mice, the use of toxic liposomes is not necessary [35]. Thus, possibly confounding toxic effects contributing to the early death of these mice is avoided. In anticipation of further studies with other epigenetic agents to induce iTreg cells, we desired to have a rapid readout for our suppressor cell assay. Therefore, we transferred a large dose of human CD25^−^ depleted human PBMC to have a rapid demise of the mice studied.

The addition of CD4^+^ cells conditioned with atRA and TGF-β, but not TGF-β by itself, delayed the onset of weight loss and extended their survival for two additional months. Other groups had reported that expanded human nTreg cells had protective effects in this mouse [35], [36], [37]. We learned that the protective effects of iTregs were at least as strong as nTregs. It is not surprising that the protective effect of these Treg cell subsets in NOG mice was not permanent. These Treg cells require human IL-2 to maintain Foxp3 expression[46], and production of this cytokine by the human cells engrafted in these mice will decrease with time. The result will be a corresponding decrease in the suppressive effects of the Treg cells.

Some workers have suggested that FOXP3 expression by TGF-β induced iTregs is only transient [47]. Others have reported that iTregs cannot suppress acute GVHD [48]. It has become evident that the methodology used to prepare mouse iTregs affects both the stability of Foxp3 expression and the protective effects of these cells *in vivo* [12], [49]. The methodology used to prepare human iTregs in this study resulted in suppressive activity that was equivalent to that of nTregs in protecting immunodeficient mice from a rapidly fatal xeno-GVHD. To address the mechanism of the protective effect of iTregs, we provide evidence that they may have suppressed the numbers of human mononuclear cells trafficking to various organs, as others have reported for expanded nTregs [36], [37].

The last and most important new finding of this study is that, in contrast to expanded nTregs, atRA-induced iTregs were resistant to the inhibitory effects of the pro-inflammatory cytokines IL-1β and IL-6 on an *in vivo* protective activity. It has become evident that Foxp3^+^ Tregs are not stable. These cytokines can down-regulate Foxp3 expression and convert these Tregs to Th17 effector cells [38], [39]. This effect can have adverse consequences in established chronic immune-mediated diseases where these cytokines are very abundant. Since retinoic acid in mice can stabilize Foxp3 [23], [24], [25] and confer T cells resistance to Th17 conversion [25], [26], [50], [51], we expected that human atRA-induced iTregs would be resistant to the inhibitory effects of IL-1β and IL-6. In mice where IL-2 and TGF-β are sufficient to induce iTregs, we found that these cytokines enabled these iTregs to be resistant to Th17 conversion by IL-6 [52].

Because of the well described protective effects of Tregs in immunologic diseases and allograft rejection, it is possible that these cells can be exploited as a therapeutic modality. Efforts are currently underway to use expanded endogenous CD4regs for this purpose. However, because of the small numbers of these cells in the blood, the technical difficulties to expand them and their instability after extended expansion [53], this procedure may be impractical for commercial development. Alternatively, personalized iTreg therapy may be more practical because: 1) large numbers of CD4^+^CD45RA^+^ cells can be obtained following pheresis; 2) the procedures to obtain these cells would utilize present methodologies used to isolate stem cells; 3) it is likely that these cells will have proliferative potential *in vivo* following transfer, and 4) atRA induced iTregs are resistant to Th17 conversion. Thus, the generation of Tregs *ex-vivo* is a promising therapeutic strategy to treat autoimmune diseases and prevent allograft rejection.

## Materials and Methods

### Mice

NOD/scid/IL2r common γ chain^−/−^ (NOG) mice were obtained from Jackson Laboratory (Bar Harbor, ME). The mice were bred and housed under specific pathogen-free conditions in microisolator cages and given unrestricted access to autoclaved food and sterile water. Animals of both sexes were used for experiments at 8–12 weeks of age. The mice received a single dose of 200 cGy gamma irradiation from a linear accelerator before injection of human PBMC on the same day. Some mice were irradiated but did not receive human PBMC. All experiments were performed according to the guidelines of the Institutional Animal Committee of the University of Southern California.

### Monoclonal antibodies and cytokines used

The following FITC, PE or Cyc conjugated human antibodies were used for flow cytometric analysis: CD4 (RM4-5), CD25 (PC61), CD45RA (L48), CD45RO (UCHL1), CD122 (Mik-β3), CD127 (hIL-7R-M21), CD103 (Ber-ACT8), CD28 (CD28.2), CCR4 (1G1), CCR7 (3D12), CTLA-4 (BNI3), PD-1 (MIH4), Foxp3 (FJK-16), TNF-αRII (2B7/97) and TGF-β (4E8). All reagents were purchased from BD PharMingen (San Diego, CA) and eBiosciences (San Diego, CA). Other agents purchased included: OKT3 from Ortho Biotech Products (Bridgewater, NJ), all-trans retinoic acid (atRA) and RPMI medium from Sigma-Aldrich (St. Louis, MO), Biotin-conjugated anti-GITR, recombinant human TGF-β1 and IL-2 from R&D Systems Inc. (Minneapolis, MN), IL-1β and IL-6 from HumanZyme (Chicago, IL), anti-human CD3/CD28-conjugated Dynabeads and carboxyfluorescein succinimidyl ester (CFSE), and AIM-V serum-free medium from Invitrogen (Carlsbad, CA), rapamycin from Calbiochem® EMD Chemicals (Gibbstown, NJ).

### Isolation of human nTregs and generation of human iTreg cells *ex vivo*

PBMC were prepared from heparinized venous blood of healthy adult volunteers by Ficoll-Hypaque density gradient centrifugation. All protocols that involved human blood donors were approved by the IRB at the University of Southern California. T cells were prepared by negative selection as described previously to a purity of >95% [8]. The CD4^+^CD25^high^ cells were obtained by fluorescence-activated cell sorting and FOXP3 expressed by these cells was >90%. These endogenous Treg cells are a mixture of nTregs and iTregs induced in vivo, but for simplicity they will be called nTregs. They were expanded for two weeks by activation with anti-CD3/CD28 beads in the presence of IL-2 (300 U/ml) and rapamycin (100 nM). CD4^+^CD45RA cells were isolated from the CD4^+^CD25^−^ cells by negative selection and activated with anti-human CD3/CD28 beads 1∶10 (one bead to 10 cells) in AIM-V serum-free medium containing Hepes buffer (10 mM), sodium pyruvate (1 mM), glutamine, non-essential amino acids and penicillin and streptomycin. This complete medium was supplemented with IL-2 (50–100 U/ml) ± TGF-β1 (5 ng/ml) ± atRA (100 nM). The dose of TGF-β1 was determined from testing concentrations from 1–20 ng/ml, and atRA from testing 0.01–1000 nM. The populations studied were 1) naive CD4^+^CD45RA^+^ cells activated with IL-2 (T_med_): 2) CD4^+^ cells activated with IL-2 and TGF-β (T-_TGF-_β); 3) CD4+ cells activated with IL-2 and atRA (T-_atRA_); and 4) CD4^+^ cells activated with IL-2, TGF-β and atRA (T-_atRA/TGF-_β or iTreg cells). The cells were stimulated for 5 days in 24 or 48 well plates, washed, and transferred to new wells with fresh culture medium containing IL-2 (50–100 U/m) unless stated otherwise. Depending upon cell density, they were split and fresh culture medium with the corresponding additives replaced every 3 days.

### Treatment of iTregs and nTregs with IL-1β and IL-6

Naïve CD4^+^ cells that had been stimulated with anti-CD3/28 beads with IL-2 (50 U/ml), TGF-β and atRA for 7 days and nTregs that had been expanded with anti-CD3/28 beads and IL-2 (300 U/ml) for 1 to 2 weeks were prepared. Foxp3 expressed by iTregs was between 70 to 75%% and expanded nTregs was between 75 to 80%. After the cells were harvested, the beads were removed and each preparation restimulated with anti-CD3/28 beads (1∶10), IL-2 (12.5 U/ml), IL-1β and IL-6 (20 ng/ml) for 3 days. This low dose of IL-2 was chosen since others have reported this amount is required for IL-1β to convert nTregs to Th17 cells [39]. High dose IL-2 was avoided because this dose would stabilize Foxp3 expression [54], and thus mask the inhibitory effects of IL-1β and IL-6 on Foxp3^+^ Treg cells. Neither additional atRA nor TGF-β was added to the iTregs.

### Suppressive assays of CD4^+^ Treg cells *in vitro* and *in vivo*

The T cells were labeled with CFSE as previously described[8]. Various ratios of CD4^+^ conditioned T cells were added to CD25 depleted T cells (T responder cells) and stimulated with soluble OKT3 (20 ng/ml) for 96 hours in the presence of irradiated (30 Gy) non-T cells (1∶1 ratio). Cell division was monitored by levels of CFSE dilution. The model to assess suppressor activity in vivo was to protect mice from a rapidly fatal GVHD as described previously[36]. Twenty million/0.2 ml CD25 depleted human PBMC were injected IV into NOG mice sublethally irradiated with 200cGy. Five×10^6^ conditioned Treg or T control CD4+ cell subsets stimulated with or without IL-1β and IL-6 were mixed with 20×10^6^ PBMC and transferred to the mice. Other mice received 5×10^6^ naive CD4^+^CD45RA^+^ cells + PBMC. The animals were examined and weighed every two days for evidence of GVHD. The mice were bled 2 weeks after cell injection and human IgG in recipient sera was measured by an ELISA using a human immunoglobulin assay kit (Bethyl, Montgomery, IL).

### Histological examination of human mononuclear cell engraftment in NOG mice

Since animals that received PBMC ± non-Treg CD4^+^ cells died between 14 and 16 days, another series of mice given similar cells were all sacrificed at 15 days for a comparative histologic evaluation of mice that received Treg or non-Treg cells. Peripheral blood obtained by cardiac puncture, spleen, liver kidney, lung, intestine and skin were harvested from recipient mice. Samples were either fixed in formalin for histologic analysis, or collagenase digested and subjected to Ficoll/Hypaque centrifugation to study engrafted human mononuclear cells. After formaldehyde fixation, paraffin sections were stained with hematoxylin and eosin. The sections were scanned with a Duoscan T2000XL microscope, and photos were taken with a Nikon 80i digital Camera.

### Flow cytometric analysis

Single cells suspensions were stained with conjugated anti-human lymphocyte antibodies indicated above. Percentages of human CD4, CD8, NK and B cells in mouse tissues were determined by gating on human anti-CD45^+^ cells. All analytic flow cytometry was done on a modified dual laser LSRScan (BD Immunocytometry Systems, San Diego, CA). For the membrane bound TGF-β staining, each previously primed CD4+ cell subset was restimulated with anti-CD3/CD28 beads (1∶1) for 72 h, and stained with anti-TGF-β or isotype control at 37°C for 4 h.

### Statistical analysis

Differences in animal Kaplan-Meier survival curves were analyzed by the log-rank test. Differences in proliferation and phenotypes of T cells, FOXP3 expression, and serum IgG levels were analyzed using the 2-tailed Student t test using Prism 4 software (San Diego, CA).

## Supporting Information

Figure S1 **Stability of homing receptors on Tregs induced with atRA and TGF-β.** AtRA/TGF-β-iTregs and expanded nTregs were rested for 2 days and restimulated with anti-CD3/28 beads for 3 days. The cells were then stained for CCR4 and CCR7 and examined by flow cytometry for expression of these chemokine receptors. This result was observed in three separate experiments.
(TIF)

Figure S2 **Suppressive activity by Tregs induced with atRA and TGF-β can be abolished by anti-TGF-β antibody.** The various primed T cell subsets shown were tested in an *in vitro* suppressive assay as described in Figure 5. In this experiment the suppressive activity was abolished by anti-TGF-β.
(TIF)

Figure S3 **Engraftment human cells in NOG mice 15 days after transfer CD4+ cells activated with TGF-β and expanded nTreg cells.** Hematoxylin and eosin sections of organs from the mice indicated were prepared as described above and compared with sections from control mice injected with PBS. The result shown is representative of studies in three mice.
(TIF)

Figure S4 **Effect of IL-1β and IL-6 on the phenotype of iTregs and expanded nTregs.** A) Histograms of Foxp3 expression by iTregs and nTregs at the conclusion of the primary cultures and other markers after the Tregs were re-stimulated for 3 days± IL-1β and IL-6. This experiment was repeated twice with similar results.
(TIF)

## References

[1] Tang Q, Bluestone JA (2008). The Foxp3+ regulatory T cell: a jack of all trades, master of regulation.. Nat Immunol.

[2] Miyara M, Yoshioka Y, Kitoh A, Shima T, Wing K (2009). Functional delineation and differentiation dynamics of human CD4+ T cells expressing the FoxP3 transcription factor.. Immunity.

[3] Bennett CL, Christie J, Ramsdell F, Brunkow ME, Ferguson PJ (2001). The immune dysregulation, polyendocrinopathy, enteropathy, X-linked syndrome (IPEX) is caused by mutations of FOXP3.. Nat Genet.

[4] Cosmi L, Liotta F, Lazzeri E, Francalanci M, Angeli R (2003). Human CD8+CD25+ thymocytes share phenotypic and functional features with CD4+CD25+ regulatory thymocytes.. Blood.

[5] Singh RP, La Cava A, Wong M, Ebling F, Hahn BH (2007). CD8+ T cell-mediated suppression of autoimmunity in a murine lupus model of peptide-induced immune tolerance depends on Foxp3 expression.. J Immunol.

[6] Miyara M, Wing K, Sakaguchi S (2009). Therapeutic approaches to allergy and autoimmunity based on FoxP3+ regulatory T-cell activation and expansion.. J Allergy Clin Immunol.

[7] Thornton AM, Korty PE, Tran DQ, Wohlfert EA, Murray PE (2010). Expression of Helios, an Ikaros transcription factor family member, differentiates thymic-derived from peripherally induced Foxp3+ T regulatory cells.. J Immunol.

[8] Horwitz DA, Zheng SG, Gray JD (2008). Natural and TGF-beta-induced Foxp3(+)CD4(+) CD25(+) regulatory T cells are not mirror images of each other.. Trends Immunol.

[9] Curotto de Lafaille MA, Lafaille JJ (2009). Natural and adaptive foxp3+ regulatory T cells: more of the same or a division of labor?. Immunity.

[10] Liu W, Putnam AL, Xu-Yu Z, Szot GL, Lee MR (2006). CD127 expression inversely correlates with FoxP3 and suppressive function of human CD4+ T reg cells.. J Exp Med.

[11] Godebu E, Summers-Torres D, Lin MM, Baaten BJ, Bradley LM (2008). Polyclonal adaptive regulatory CD4 cells that can reverse type I diabetes become oligoclonal long-term protective memory cells.. J Immunol.

[12] Selvaraj RK, Geiger TL (2008). Mitigation of Experimental Allergic Encephalomyelitis by TGF-{beta} Induced Foxp3+ Regulatory T Lymphocytes through the Induction of Anergy and Infectious Tolerance.. J Immunol.

[13] Aricha R, Feferman T, Fuchs S, Souroujon MC (2008). Ex vivo generated regulatory T cells modulate experimental autoimmune myasthenia gravis.. J Immunol.

[14] Horwitz DA (2008). Regulatory T cells in systemic lupus erythematosus: past, present and future.. Arthritis Res Ther.

[15] Zheng SG, Gray JD, Ohtsuka K, Yamagiwa S, Horwitz DA (2002). Generation ex vivo of TGF-beta-producing regulatory T cells from CD4+CD25- precursors.. J Immunol.

[16] Zheng SG, Wang JH, Koss MN, Quismorio F, Gray JD (2004). CD4+ and CD8+ regulatory T cells generated ex vivo with IL-2 and TGF-beta suppress a stimulatory graft-versus-host disease with a lupus-like syndrome.. J Immunol.

[17] Zheng SG, Wang JH, Gray JD, Soucier H, Horwitz DA (2004). Natural and induced CD4+CD25+ cells educate CD4+CD25- cells to develop suppressive activity: the role of IL-2, TGF-beta, and IL-10.. J Immunol.

[18] Rao PE, Petrone AL, Ponath PD (2005). Differentiation and expansion of T cells with regulatory function from human peripheral lymphocytes by stimulation in the presence of TGF-{beta}.. J Immunol.

[19] Gavin MA, Torgerson TR, Houston E, DeRoos P, Ho WY (2006). Single-cell analysis of normal and FOXP3-mutant human T cells: FOXP3 expression without regulatory T cell development.. Proc Natl Acad Sci U S A.

[20] Tran DQ, Ramsey H, Shevach EM (2007). Induction of FOXP3 expression in naive human CD4+FOXP3 T cells by T-cell receptor stimulation is transforming growth factor-beta dependent but does not confer a regulatory phenotype.. Blood.

[21] Horwitz DA, Zheng SG, Wang J, Gray JD (2008). Critical role of IL-2 and TGF-beta in generation, function and stabilization of Foxp3(+)CD4(+) Treg.. Eur J Immunol.

[22] Baecher-Allan C, Brown JA, Freeman GJ, Hafler DA (2001). CD4+CD25high regulatory cells in human peripheral blood.. J Immunol.

[23] Mucida D, Pino-Lagos K, Kim G, Nowak E, Benson MJ (2009). Retinoic acid can directly promote TGF-beta-mediated Foxp3(+) Treg cell conversion of naive T cells.. Immunity.

[24] Hill JA, Hall JA, Sun CM, Cai Q, Ghyselinck N (2008). Retinoic acid enhances Foxp3 induction indirectly by relieving inhibition from CD4+CD44hi Cells.. Immunity.

[25] Nolting J, Daniel C, Reuter S, Stuelten C, Li P (2009). Retinoic acid can enhance conversion of naive into regulatory T cells independently of secreted cytokines.. J Exp Med.

[26] Kang SG, Lim HW, Andrisani OM, Broxmeyer HE, Kim CH (2007). Vitamin A metabolites induce gut-homing FoxP3+ regulatory T cells.. J Immunol.

[27] Wang J, Huizinga TW, Toes RE (2009). De novo generation and enhanced suppression of human CD4+CD25+ regulatory T cells by retinoic acid.. J Immunol.

[28] Zheng SG, Wang J, Wang P, Gray JD, Horwitz DA (2007). IL-2 is essential for TGF-beta to convert naive CD4+CD25- cells to CD25+Foxp3+ regulatory T cells and for expansion of these cells.. J Immunol.

[29] Chen X, Subleski JJ, Kopf H, Howard OM, Mannel DN (2008). Cutting edge: expression of TNFR2 defines a maximally suppressive subset of mouse CD4+CD25+FoxP3+ T regulatory cells: applicability to tumor-infiltrating T regulatory cells.. J Immunol.

[30] Nakamura K, Kitani A, Fuss I, Pedersen A, Harada N (2004). TGF-beta 1 plays an important role in the mechanism of CD4+CD25+ regulatory T cell activity in both humans and mice.. J Immunol.

[31] Lim HW, Broxmeyer HE, Kim CH (2006). Regulation of trafficking receptor expression in human forkhead box P3+ regulatory T cells.. J Immunol.

[32] Schneider MA, Meingassner JG, Lipp M, Moore HD, Rot A (2007). CCR7 is required for the in vivo function of CD4+ CD25+ regulatory T cells.. J Exp Med.

[33] Iellem A, Colantonio L, D'Ambrosio D (2003). Skin-versus gut-skewed homing receptor expression and intrinsic CCR4 expression on human peripheral blood CD4+CD25+ suppressor T cells.. Eur J Immunol.

[34] Izcue A, Coombes JL, Powrie F (2006). Regulatory T cells suppress systemic and mucosal immune activation to control intestinal inflammation.. Immunol Rev.

[35] Ito R, Katano I, Kawai K, Hirata H, Ogura T (2009). Highly sensitive model for xenogenic GVHD using severe immunodeficient NOG mice.. Transplantation.

[36] Mutis T, van Rijn RS, Simonetti ER, Aarts-Riemens T, Emmelot ME (2006). Human regulatory T cells control xenogeneic graft-versus-host disease induced by autologous T cells in RAG2-/-gammac-/- immunodeficient mice.. Clin Cancer Res.

[37] Hippen KL, Harker-Murray P, Porter SB, Merkel SC, Londer A (2008). Umbilical cord blood regulatory T-cell expansion and functional effects of tumor necrosis factor receptor family members OX40 and 4-1BB expressed on artificial antigen-presenting cells.. Blood.

[38] Beriou G, Costantino CM, Ashley CW, Yang L, Kuchroo VK (2009). IL-17-producing human peripheral regulatory T cells retain suppressive function.. Blood.

[39] Koenen HJ, Smeets RL, Vink PM, van Rijssen E, Boots AM (2008). Human CD25highFoxp3pos regulatory T cells differentiate into IL-17-producing cells.. Blood.

[40] Pasare C, Medzhitov R (2003). Toll pathway-dependent blockade of CD4+CD25+ T cell-mediated suppression by dendritic cells.. Science.

[41] Austrup F, Rebstock S, Kilshaw PJ, Hamann A (1995). Transforming growth factor-beta 1-induced expression of the mucosa-related integrin alpha E on lymphocytes is not associated with mucosa-specific homing.. Eur J Immunol.

[42] Coombes JL, Siddiqui KR, Arancibia-Carcamo CV, Hall J, Sun CM (2007). A functionally specialized population of mucosal CD103+ DCs induces Foxp3+ regulatory T cells via a TGF-beta and retinoic acid-dependent mechanism.. J Exp Med.

[43] Yang ZZ, Novak AJ, Stenson MJ, Witzig TE, Ansell SM (2006). Intratumoral CD4+CD25+ regulatory T-cell-mediated suppression of infiltrating CD4+ T cells in B-cell non-Hodgkin lymphoma.. Blood.

[44] Oida T, Xu L, Weiner HL, Kitani A, Strober W (2006). TGF-beta-mediated suppression by CD4+CD25+ T cells is facilitated by CTLA-4 signaling.. J Immunol.

[45] Savage ND, de Boer T, Walburg KV, Joosten SA, van Meijgaarden K (2008). Human anti-inflammatory macrophages induce Foxp3+ GITR+ CD25+ regulatory T cells, which suppress via membrane-bound TGFbeta-1.. J Immunol.

[46] Fontenot JD, Rasmussen JP, Gavin MA, Rudensky AY (2005). A function for interleukin 2 in Foxp3-expressing regulatory T cells.. Nat Immunol.

[47] Baron U, Floess S, Wieczorek G, Baumann K, Grutzkau A (2007). DNA demethylation in the human FOXP3 locus discriminates regulatory T cells from activated FOXP3(+) conventional T cells.. Eur J Immunol.

[48] Koenecke C, Czeloth N, Bubke A, Schmitz S, Kissenpfennig A (2009). Alloantigen-specific de novo-induced Foxp3+ Treg revert in vivo and do not protect from experimental GVHD.. Eur J Immunol.

[49] Selvaraj RK, Geiger TL (2007). A kinetic and dynamic analysis of Foxp3 induced in T cells by TGF-beta.. J Immunol.

[50] Xiao S, Jin H, Korn T, Liu SM, Oukka M (2008). Retinoic acid increases Foxp3+ regulatory T cells and inhibits development of Th17 cells by enhancing TGF-beta-driven Smad3 signaling and inhibiting IL-6 and IL-23 receptor expression.. J Immunol.

[51] Zhou X, Kong N, Wang J, Fan H, Zou H (2010). Cutting Edge: All-Trans Retinoic Acid Sustains the Stability and Function of Natural Regulatory T Cells in an Inflammatory Milieu.. J Immunol.

[52] Zheng SG, Wang J, Horwitz DA (2008). Cutting Edge: Foxp3+CD4+CD25+ Regulatory T Cells Induced by IL-2 and TGF-{beta} Are Resistant to Th17 Conversion by IL-6.. J Immunol.

[53] Hoffmann P, Boeld TJ, Eder R, Huehn J, Floess S (2009). Loss of FOXP3 expression in natural human CD4+CD25+ regulatory T cells upon repetitive in vitro stimulation.. Eur J Immunol.

[54] Elias KM, Laurence A, Davidson TS, Stephens G, Kanno Y (2008). Retinoic acid inhibits Th17 polarization and enhances FoxP3 expression through a Stat-3/Stat-5 independent signaling pathway.. Blood.

