# Correction: Characterization of Protective Human CD4^+^CD25^+^ FOXP3^+^ Regulatory T Cells Generated with IL-2, TGF-β and Retinoic Acid

**DOI:** 10.1371/annotation/8f29ac57-8219-4ffd-84c9-5e6bf6dcd017

**Published:** 2011-01-07

**Authors:** Ling Lu, Xiaohui Zhou, Julie Wang, Song Guo Zheng, David A. Horwitz

Information was missing from the Funding section. The funding section should read: "This work was supported by grants from ExCell Therapeutics LLC, the Nora Eccles Treadwell Foundation, and BD Biosciences (to D.A.H.) and National Institutes of Health Grant R01 AR059103, ACR Research and Education Foundation's Within Our Reach, Arthritis Foundation, Outstanding Youth Scientist Investigator Award from National Natural Science Foundation of China (30728007) and East Shanghai Scientific Foundation (PKJ2009-Y41) (to S.G.Z).

