# Supplementary figures and images for: Characterization of Protective Human CD4+CD25+ FOXP3+ Regulatory T Cells Generated with IL-2, TGF-β and Retinoic Acid
Source: PLoS One. 2010 Dec 17;5(12):e15150. doi: 10.1371/journal.pone.0015150 (PMC3003689; doi:10.1371/journal.pone.0015150)

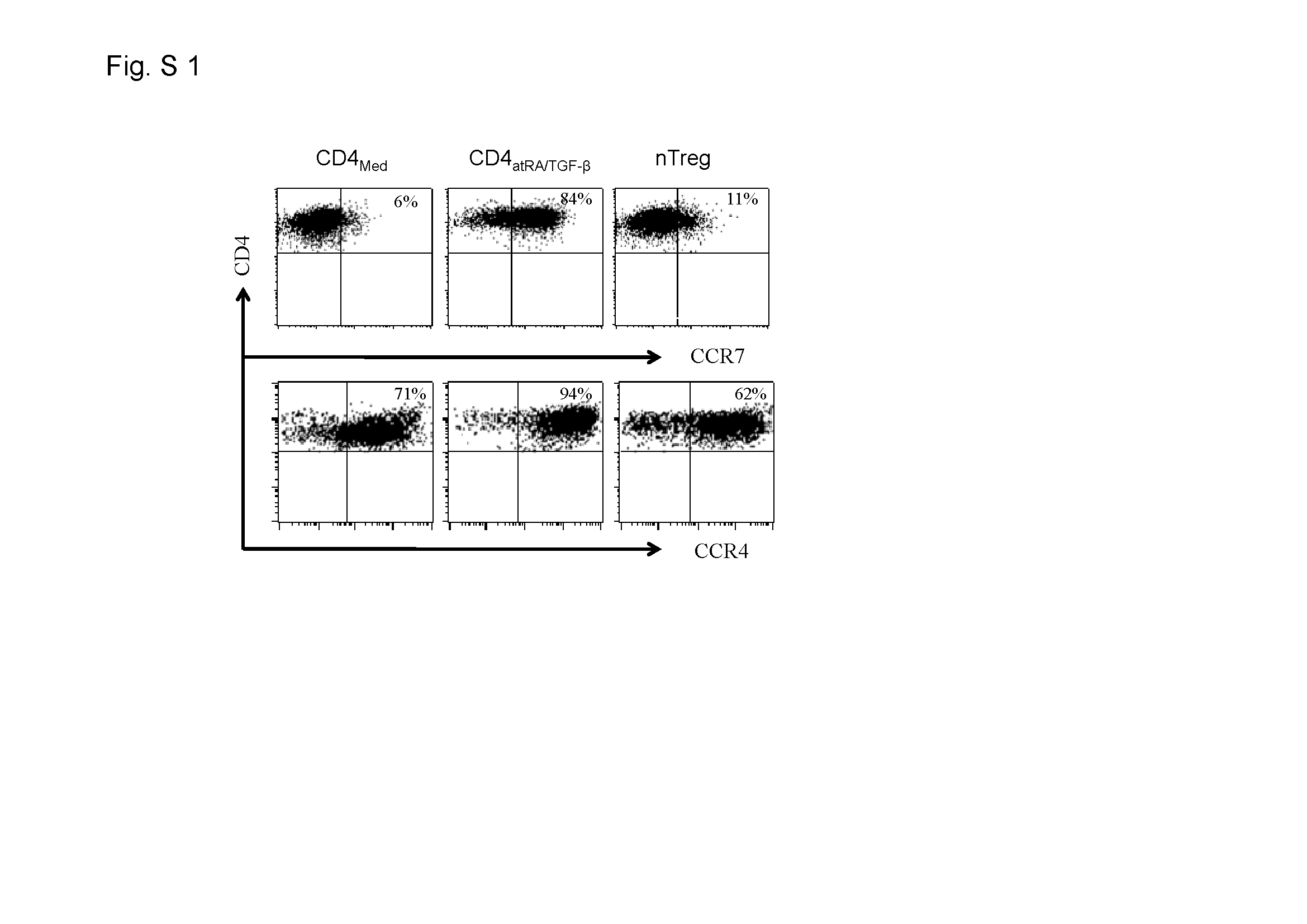

Supplement: Figure S1 — Stability of homing receptors on Tregs induced with atRA and TGF-β. AtRA/TGF-β-iTregs and expanded nTregs were rested for 2 days and restimulated with anti-CD3/28 beads for 3 days. The cells were then stained for CCR4 and CCR7 and examined by flow cytometry for expression of these chemokine receptors. This result was observed in three separate experiments. (TIF) [file pone.0015150.s001.tif]

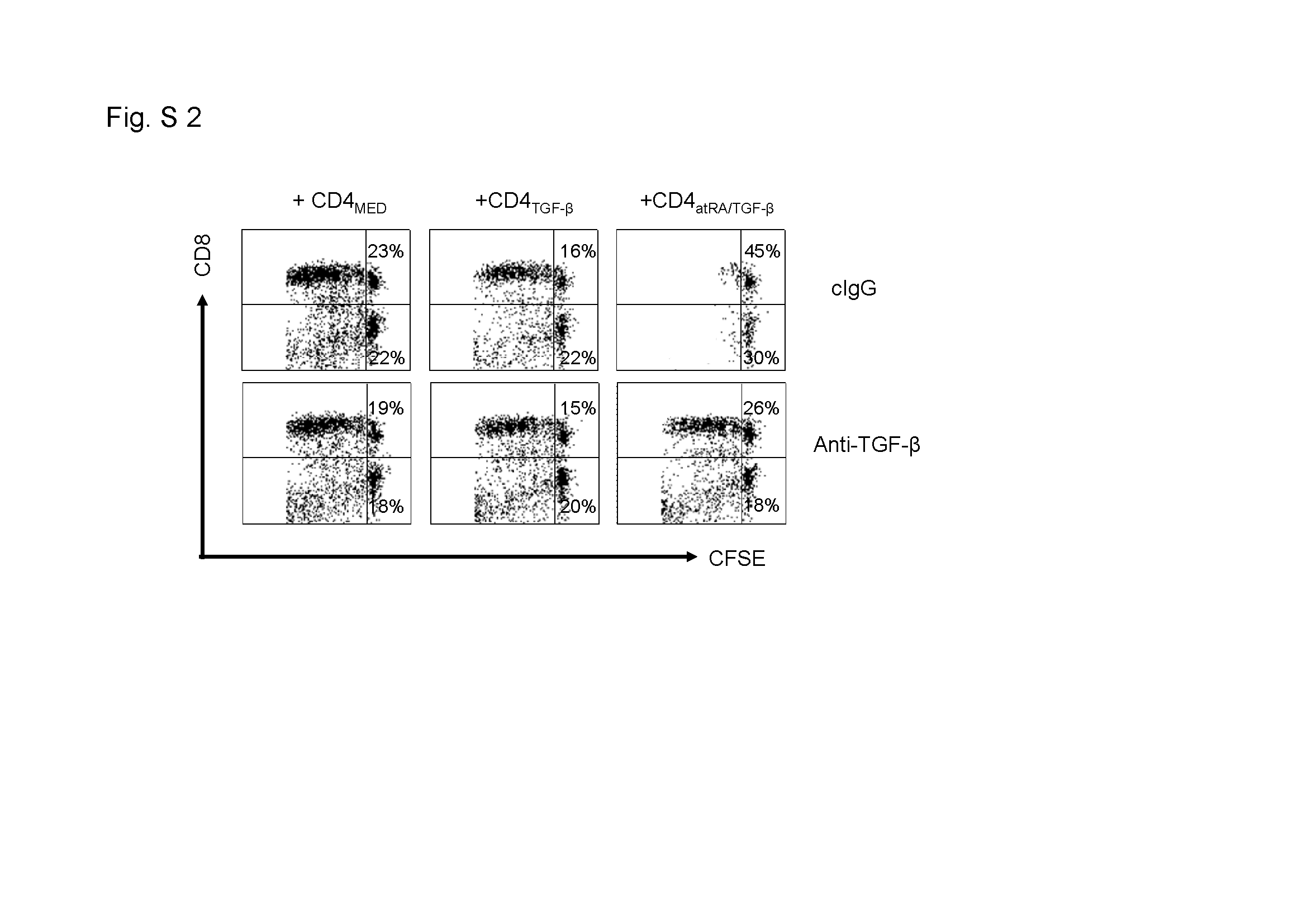

Supplement: Figure S2 — Suppressive activity by Tregs induced with atRA and TGF-β can be abolished by anti-TGF-β antibody. The various primed T cell subsets shown were tested in an in vitro suppressive assay as described in Figure 5. In this experiment the suppressive activity was abolished by anti-TGF-β. (TIF) [file pone.0015150.s002.tif]

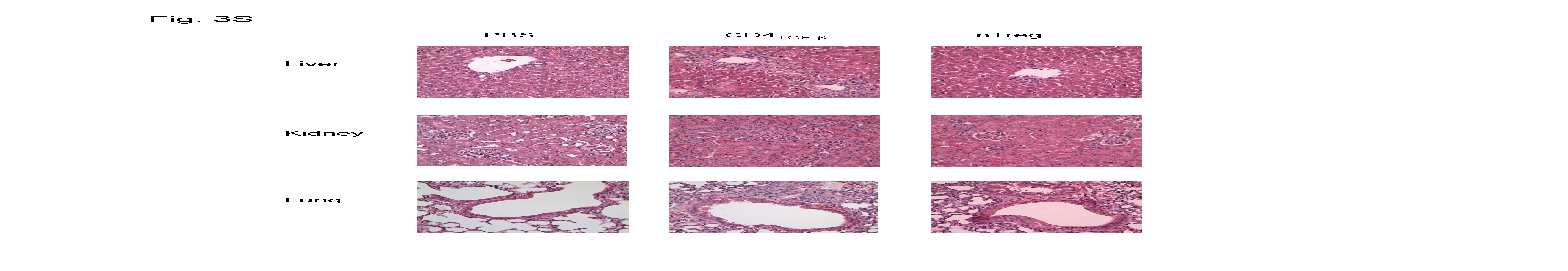

Supplement: Figure S3 — Engraftment human cells in NOG mice 15 days after transfer CD4+ cells activated with TGF-β and expanded nTreg cells. Hematoxylin and eosin sections of organs from the mice indicated were prepared as described above and compared with sections from control mice injected with PBS. The result shown is representative of studies in three mice. (TIF) [file pone.0015150.s003.tif]

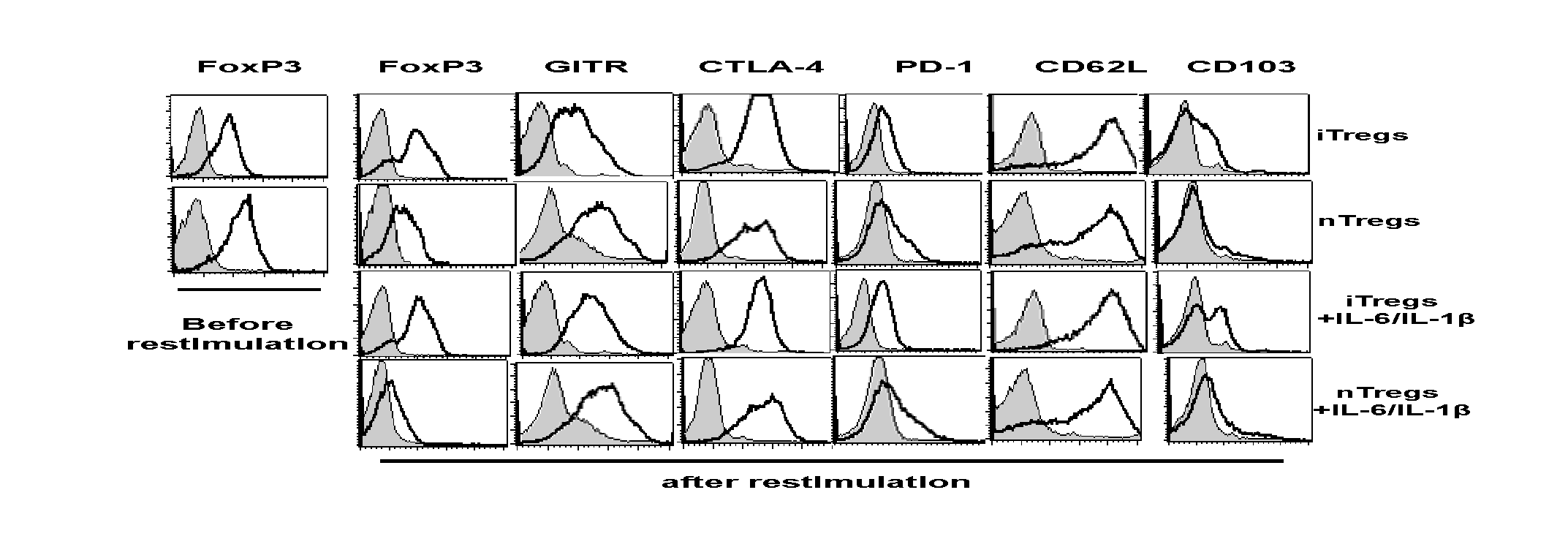

Supplement: Figure S4 — Effect of IL-1β and IL-6 on the phenotype of iTregs and expanded nTregs. A) Histograms of Foxp3 expression by iTregs and nTregs at the conclusion of the primary cultures and other markers after the Tregs were re-stimulated for 3 days± IL-1β and IL-6. This experiment was repeated twice with similar results. (TIF) [file pone.0015150.s004.tif]
